# Use, Potential Use, and Awareness of the 988 Suicide and Crisis Lifeline by Level of Psychological Distress

**DOI:** 10.1001/jamanetworkopen.2023.41383

**Published:** 2023-10-31

**Authors:** Jonathan Purtle, Anna-Michelle Marie McSorley, Abigail Lin Adera, Michael A. Lindsey

**Affiliations:** 1 Department of Public Health Policy and Management, School of Global Public Health, New York University, New York; 2 Center for Anti-racism, Social Justice, and Public Health, School of Global Public Health, New York University, New York; 3 University of Miami, Coral Gables, Florida; 4 Silver School of Social Work, New York University, New York

## Abstract

This cross-sectional study assesses variations in use, potential use, and awareness of the 988 Suicide and Crisis Lifeline among people with varying levels of psychological distress.

## Introduction

The 988 Suicide and Crisis Lifeline was launched nationally on July 16, 2022. Increases in call volume following the launch of 988 hotline^1^ and public awareness^2^ and policy maker communication^3^ about the 988 Lifeline have been documented. However, little is known about how use or awareness of the 988 Lifeline varies across populations with different levels of psychological distress. This represents an important area of study.^4^ To address this, we assessed variations in use, potential use, and awareness of the 988 Lifeline among people with varying levels of psychological distress.

## Methods

This cross-sectional study was approved by the New York University institutional review board and followed the Strengthening the Reporting of Observational Studies in Epidemiology (STROBE) reporting guideline. Informed consent was obtained by Ipsos when participants joined the KnowledgePanel.

A nationally representative web-based survey of 5058 US adults was conducted between June 9 to June 19, 2023 using the Ipsos KnowledgePanel (response rate, 55.0%). The KnowledgePanel uses address-based and probability-based sampling and survey-specific weights to produce representative estimates of the US adult population. Respondents are blind to survey topics when recruited, reducing the risk of selection bias.

The independent variable was a 3-level ordinal variable of past 30-day psychological distress, assessed using Kessler K6 Scale (K6).^5^ The K6 has total classification accuracy of 0.92 at a scoring cut point of 13 or more (serious distress)^5^ and 0.74 at a cut point of 5 or more (moderate distress).^6^ K6 scores (Cronbach α = .93) were calculated for 97.7% of respondents, who were categorized as having serious, moderate, or no distress based on established cut points.^5,6^

The dependent variables were whether respondents had: (1) heard of 988 (yes/no); (2) had used 988 on behalf of themselves (yes/no); and (3) their likelihood of using 988 in the future if they or a loved one were experiencing a crisis or suicidality (dichotomized as very likely [yes/no], 6 to 7 on 7-point scale). The verbatim items are available in eAppendix in Supplement 1.

Descriptive statistics characterized dependent variables with 95% CIs, stratified by distress level. Multivariable logistic regression assessed significant associations between distress levels and dependent variables, adjusting for demographic covariates. Missing data were excluded from analysis. Tests were 2-tailed, statistical significance was set at *P* < .05, and analyses were conducted in July 2023. Analyses were conducted with SPSS Statistics 28.0 (IBM).

## Results

This study included 4942 respondents who completed all K6 items. Of these respondents, 2521 (51.0%) identified as female, 2421 (49.0%) identified as male, and the mean (SD) age was 48.1 (17.7) years. The serious distress category included 388 respondents (7.9%), moderate distress included 1006 respondents (20.4%), and no distress included 3548 respondents (71.8%). Respondents with serious distress were significantly more likely to have heard of the 998 Lifeline than those with no distress (184 of 388 [47.4%]; 95% CI, 42.4%-52.4% vs 1422 of 3548 [40.4%]; 95% CI; 38.8%-42.1%; *P* = .007) (Table 1). The proportion who had used the 988 Lifeline was substantially larger among respondents with serious distress (23 of 388 [6.0%]; 95% CI, 3.6%-8.3%; *P* < .001) than those with moderate distress (10 of 1006 [1.0%]; 95% CI, 0.4%-1.6%; *P* < .001) and no distress (6 of 3548 [0.2%]; 95% CI, 0%-0.3%; *P* < .001). However, a slightly smaller proportion of respondents with serious distress than no distress reported being very likely to use 988 in the future (86 of 388 [22.3%]; 95% CI, 18.1%-26.4% vs 930 of 3548 [26.2%]; 95% CI, 24.8%-27.7%; *P* = .08). Among the 23 respondents with serious distress who had used the 988 Lifeline, only 7 (29.0%) were very likely to use it in the future.

**Table 1.** Awareness, Past Use, and Potential Future Use of 988 Suicide and Crisis Lifeline Among US Adults, Stratified by Past 30-Day Psychological Distress Status, June 2023 (N = 4942)

Psychological distress category	Has heard of 988 Lifeline		Has used 988 Lifeline on behalf of themselves		Very likely to use 988 Lifeline in the future if self or loved one were experiencing a mental health crisis or suicidality^a^	
	% (95% CI)	*P* value	% (95% CI)	*P* value	% (95% CI)	*P* value
All	41.8 (40.5-43.2)	NA	0.8 (0.5-1.0)	NA	24.6 (23.4-25.8)	NA
Psychological distress, past 30-d (K6 score)						
Serious (≥13)	47.4 (42.4-52.4)	.007	6.0 (3.6-8.3)	<.001	22.3 (18.1-26.4)	.08
Moderate (5-12)	45.0 (41.9-48.1)	<.001	1.0 (0.4-1.6)	<.001	20.7 (18.2-23.2)	<.001
None (≤11)	40.4 (38.8-42.1)	NA	0.2 (0-0.3)	NA	26.2 (24.8-27.7)	NA

Abbreviation: NA, not applicable.

^a^ Rating of 6 or 7 on a 7-point scale.

Adjusting for demographics, having serious distress (adjusted odds ratio [AOR], 1.45; 95% CI, 1.16-1.82; *P* = .001) or moderate distress (AOR, 1.27; 95% CI, 1.09-1.47; *P* = .002) was significantly associated with higher odds of having heard of the 988 Lifeline compared with respondents with no distress (Table 2). Having serious distress was independently associated with over 30 times higher odds (AOR, 31.96; 95% CI, 12.01-85.08; *P* < .001) of having used the 988 Lifeline and having moderate distress was associated with 5 times higher odds (AOR, 5.15; 95% CI, 1.78-14.89; *P* = .003).

**Table 2.** Adjusted Associations Between Awareness, Past Use, and Potential Future Use of 988 Suicide and Crisis Lifeline and Past 30-Days Psychological Distress Status (US Adults, June 2023, N = 4942)

Psychological distress category	Model 1: has heard of 988 Lifeline		Model 2: has used 988 Lifeline on behalf of themselves		Model 3: very likely to use 988 Lifeline in the future if self or loved one were experiencing a mental health crisis or suicidality^a^	
	AOR (95% CI)	*P* value	AOR (95% CI)	*P* value	AOR (95% CI)	*P* value
Psychological distress, past 30 d (K6 score)						
Serious (≥13)	1.45 (1.16-1.82)	.001	31.96 (12.01-85.08)	<.001	0.91 (0.7-1.18)	.49
Moderate (5-12)	1.27 (1.09-1.47)	.002	5.15 (1.78-14.89)	.003	0.76 (0.63-0.9)	.002
None (≤11)	1 [Reference]	NA	1 [Reference]	NA	1 [Reference]	NA
Age, y						
18-29	1.78 (1.49-2.11)	<.001	9.62 (1.51-61.28)	.02	0.64 (0.53-0.78)	<.001
30-44	1.1 (0.94-1.29)	.25	3.53 (0.53-23.76)	.19	0.6 (0.5-0.72)	<.001
45-59	1 (0.85-1.17)	.96	3.98 (0.57-27.61)	.16	0.78 (0.66-0.93)	.006
≥60	1 [Reference]	NA	1 [Reference]	NA	1 [Reference]	NA
Income, $						
<10 000	0.53 (0.36-0.77)	.001	0.3 (0.04-2.12)	.23	0.74 (0.48-1.15)	.18
10 000-24 999	0.63 (0.48-0.81)	<.001	0.65 (0.2-2.1)	.47	0.92 (0.69-1.22)	.55
25 000-49 999	0.87 (0.71-1.06)	.17	0.63 (0.22-1.8)	.39	1.28 (1.02-1.6)	.03
50 000-74 999	0.89 (0.74-1.09)	.26	0.45 (0.14-1.4)	.17	0.98 (0.79-1.23)	.87
75 000-99 999	0.93 (0.76-1.13)	.46	0.39 (0.09-1.64)	.20	1.12 (0.89-1.41)	.33
100 000-149 999	0.94 (0.78-1.12)	.48	0.47 (0.15-1.5)	.20	1.07 (0.87-1.31)	.51
≥150 000	1 [Reference]	NA	1 [Reference]	NA	1 [Reference]	NA
Highest education						
<High school	0.59 (0.46-0.75)	<.001	0.77 (0.23-2.54)	.66	1.11 (0.86-1.43)	.44
High school	0.8 (0.68-0.94)	.006	0.91 (0.36-2.34)	.85	0.87 (0.73-1.05)	.15
Some college	0.85 (0.72-0.99)	.04	0.71 (0.27-1.9)	.50	0.9 (0.76-1.08)	.27
Bachelor’s degree or higher	1 [Reference]	NA	1 [Reference]	NA	1 [Reference]	NA
Race or ethnicity						
Black/African American, non-Hispanic	0.73 (0.6-0.88)	.001	2.1 (0.81-5.47)	.13	1.45 (1.18-1.77)	<.001
Hispanic	0.56 (0.47-0.66)	<.001	0.93 (0.37-2.32)	.87	1.43 (1.19-1.72)	<.001
2 or more races, non-Hispanic	0.82 (0.61-1.1)	.19	1.35 (0.36-5.06)	6	0.97 (0.69-1.38)	.88
Other race, non-Hispanic	0.58 (0.44-0.76)	<.001	2.41 (0.74-7.89)	.15	0.81 (0.58-1.13)	.21
White, non-Hispanic	1 [Reference]	NA	1 [Reference]	NA	1 [Reference]	NA
Sex						
Male	1.04 (0.93-1.17)	.49	0.95 (0.48-1.85)	.87	0.77 (0.67-0.88)	<.001
Female	1 [Reference]	NA	1 [Reference]	NA	1 [Reference]	NA

Abbreviations: AOR, adjusted odds ratio; NA, not applicable.

^a^ Rating of 6 or 7 on a 7-point scale. Multivariable logistic regression models. Adjusted for age, sex, race or ethnicity, income, and education.

## Discussion

In this cross-sectional study, use and awareness of the 988 Lifeline was significantly higher among individuals with serious and moderate psychological distress. Approximately 1 in 20 respondents with serious distress had used the 988 Lifeline, but only about one-third of these users were very likely to use it in the future.

This study had limitations, such as the representativeness of the sample. Despite using address-based and probability-based sampling and survey-specific weights, these results may not be generalizable to all US adults. Additionally, 988 Lifeline implementation has varied between states and state-level analyses were not conducted. Despite these limitations, there is a need for more research about satisfaction with the 988 Lifeline among people with serious distress and the extent to which the 988 Lifeline, and the resources it connects users to, meets their needs.

## Notes

Supplement 1. **eAppendix.** Survey Questions

Supplement 2.
Data Sharing Statement
