# Supplementary material for: Use, Potential Use, and Awareness of the 988 Suicide and Crisis Lifeline by Level of Psychological Distress
Source: JAMA Netw Open. 2023 Oct 31;6(10):e2341383. doi: 10.1001/jamanetworkopen.2023.41383 (PMC10618841; doi:10.1001/jamanetworkopen.2023.41383)
Supplement: Supplement 1. — eAppendix. Survey Questions [file jamanetwopen-e2341383-s001.pdf]

Supplemental Online Content

Purtle J, McSorley AM, Adera AL, Lindsey MA . Variation in use, potential use, and awareness of the 988 suicide and crisis lifeline by level of psychological distress. *JAMA Netw Open*. 2023;6(10):e2341383. doi:10.1001/jamanetworkopen.2023.41383

eAppendix. Survey Questions

This supplemental material has been provided by the authors to give readers additional information about their work.

© 2023 Purtle J et al. *JAMA Network Open.*

eAppendix. Survey Questions

Wording of Survey Items (bolding emphasis included)

"988" was nationally launched as the three-digit dialing code for the 988 Suicide & Crisis Lifeline in July 2022. Please select **all that apply to you** as it relates to the 988 Lifeline.

- I have heard of the 988 Lifeline prior to this survey*
- I have **not** heard of the 988 Lifeline prior to this survey
- I have called texted, and/or chatted the 988 Lifeline on behalf of **my self***
- I have called texted, and/or chatted the 988 Lifeline on behalf of **a loved one**

Please rate, on a scale of 1-7, the likelihood of you reaching out to each of the following sources if you or a loved one were experiencing a mental health crisis or thoughts of suicide (1= Very unlikely, 7= Very likely)?

- 988 Lifeline*
- A crisis line **other than** the 988 Lifeline (e.g., Trevor Project, Crisis Text Line)
- A psychologist, psychiatrist, social worker, or other mental health professional
- A friend or family member
- Someone in my religious network

** Used in current analysis*

© 2023 Purtle J et al. *JAMA Network Open.*
