# Supplementary material for: Use, Potential Use, and Awareness of the 988 Suicide and Crisis Lifeline by Level of Psychological Distress
Source: JAMA Netw Open. 2023 Oct 31;6(10):e2341383. doi: 10.1001/jamanetworkopen.2023.41383 (PMC10618841; doi:10.1001/jamanetworkopen.2023.41383)
Supplement: Supplement 2. — Data Sharing Statement [file jamanetwopen-e2341383-s002.pdf]

Data Sharing Statement

Purtle. Use and Awareness of the 988 Suicide and Crisis Lifeline by Level of Psychological Distress. *JAMA Netw Open*. Published October 31, 2023.
doi:10.1001/jamanetworkopen.2023.41383

Data
Data available: No
